# Neonatal hearing screening - does failure in TEOAE screening matter when the AABR test is passed?

**DOI:** 10.1007/s00405-023-08250-z

**Published:** 2023-10-13

**Authors:** Donata Gellrich, Moritz Gröger, Matthias Echternach, Katharina Eder, Patrick Huber

**Affiliations:** 1 Department of Otorhinolaryngology, Head and Neck Surgery, Division of Phoniatrics and Pediatric Audiology, University Hospital, Ludwig-Maximilians-University Munich (LMU), Marchioninistr. 15, 81377 Munich, Germany; 2 Department of Pediatric Audiology, Kbo-Kinderzentrum München Gemeinnützige GmbH, Heiglhofstr. 65, 81377 Munich, Germany

**Keywords:** Newborn, Hearing screening, TEOAE, AABR, Discordance, Hearing impairment

## Abstract

**Purpose:**

Newborns who fail the transient evoked otoacoustic emissions (TEOAE) but pass the automatic auditory brainstem response (AABR) in universal newborn hearing screening (UNHS), frequently have no further diagnostic test or follow-up. The present study aimed to investigate whether hearing loss might be missed by ignoring neonatal TEOAE failure in the presence of normal AABR.

**Methods:**

A retrospective analysis was conducted in newborns presenting between 2017 and 2021 to a tertiary referral centre due to failure in the initial UNHS. The main focus was on infants who failed TEOAE tests, but passed AABR screening. The clinical characteristics and audiometric outcomes were analysed and compared with those of other neonates.

**Results:**

Among 1,095 referred newborns, 253 (23%) failed TEOAE despite passing AABR screening. Of the 253 affected infants, 154 returned for follow-up. At 1-year follow-up, 46 (28%) achieved normal audiometric results. 32 (21%) infants had permanent hearing loss (HL) confirmed by diagnostic ABR, 58 (38%) infants had HL solely due to middle ear effusion (MEE), and for 18 (12%) infants HL was suspected without further differentiation. The majority of permanent HL was mild (78% mild vs. 13% moderate vs. 9% profound). The rate of spontaneous MEE clearance was rather low (29%) leading to early surgical intervention in 36 children. The profile of the risk factors for hearing impairment was similar to that of newborns with failure in both, TEOAE and AABR; however, there was a stronger association between the presence of risk factors and the incidence of HL (relative risk 1.55 vs. 1.06; odds ratio 3.61 vs. 1.80).

**Conclusion:**

In newborns, the discordance between a “refer” in TEOAE and a “pass” in AABR screening is associated with a substantial prevalence of hearing impairment at follow-up, especially in the presence of risk factors.

## Introduction

Congenital hearing loss is one of the most common chronic pathologies in newborns with a prevalence of 1–2 per 1000 neonates in developed countries [1]. As a good hearing function is crucial for normal speech development, hearing impairment in early childhood has a high impact on children’s cognitive, emotional and social development. Therefore, early detection and treatment of hearing impairments are essential. In many countries, universal newborn hearing screening (UNHS) is nowadays implemented as part of standard neonatal care. Currently, two methods are internationally recommended as screening technologies: detection of transient evoked otoacoustic emissions (TEOAE) which record the response of the cochlear outer hair cells to a click or tone-burst stimulus, and automatic auditory brainstem response (AABR) detection which records neurophysiological responses from the brainstem to acoustic stimuli and, therefore, reflects the function of the cochlea, auditory nerve and brainstem. Both methods have high sensitivity and specificity, provided that ideal conditions for measurement such as experienced screening staff and a quiet setting are available [2].

Although AABR screening is associated with a lower referral rate than TEOAE screening [2, 3], neonates without an increased risk of hearing loss are often initially screened with TEOAE owing to the simplicity and rapidness of measurement compared to AABR. AABR screening is recommended for newborns with an increased risk of hearing impairment, [4, 5].

Consistent with the recent recommendation of the WHO [5], many countries pursue a two-stage screening protocol which combines both, TEOAE and AABR screening. In Germany, where the presented data were collected, a two-step UNHS protocol has been established [6]: in the first 3 days after birth a TEOAE or AABR screening is conducted. In newborns at an increased risk of hearing impairment, AABR-measurement is mandatory. In case of failure in the initial TEOAE or AABR screening, a control by AABR screening is carried out within a few days, at the latest on the 14th day after birth. If the infant passes this control AABR screening, no further follow-up is scheduled according to the protocol. In case the infant fails the control AABR screening, further audiometric diagnostics by a paediatric audiologist are obligatory and their completion is tracked by a governmental agency [6].

In children who fail TEOAE, but pass the subsequent AABR screening at 35 dB normalised hearing level (nHL), no further diagnostic test or follow-up is recommended according to the German UNHS protocol and the protocols of other countries, such as the two-tier screening proposed by the American Speech-Language-Hearing Association [7].

As a stimulus level of 35 dB nHL is applied in AABR to obtain the result “pass” or “refer”, mild forms of congenital hearing loss (HL) might only become apparent by absent TEOAE. Therefore, mild forms of congenital HL could be missed by UNHS protocols such as the German UNHS protocol which disregards the absence of TEOAE in the presence of a passed AABR screening. In the 1990s, the UNHS was designed to identify moderate, severe, and profound HL. However, growing evidence suggests that even mild HL is associated with delays in speech development and lower academic education [8, 9]. Therefore, the median time-to-diagnosis and time-to-treatment should be reduced even in patients with mild HL. Even unilateral mild HL should not be dismissed because it impairs sound localisation [10], speech perception in noise, and quality of life [11].

The present study aimed to investigate the number of neonates who passed the AABR screening test despite the absence of TEOAE and exhibit a hearing impairment after 1 year or later. Furthermore, the analysis had the purpose to identify distinguishing features which might help to predict a hearing impairment in newborns who fail the TEOAE and pass the AABR screening.

## Methods

We conducted a retrospective analysis of children who were referred to our tertiary referral centre between 01/2017 and 12/2021 due to failure in the initial UNHS.

All children underwent a full audiometric screening procedure of bilateral AABR (MAICO BERAphone, MAICO Diagnostics, Berlin, Germany), TEOAE, distortion product otoacoustic emissions (MADSEN Capella, GN Otometrics GmbH, Münster, Germany), and tympanometry (Auritec Medizindiagnostische Systeme, Hamburg, Germany) during their first consultation.

According to the screening results, a further follow-up was scheduled: In case of a “refer” result in AABR, chirp-evoked auditory brainstem responses for air conduction (ABR) at 1, 2 and 4 kHz and click-evoked bone conduction ABR were measured (hereinafter referred to as ‘diagnostic ABR’). If the newborns passed the AABR screening, but failed the TEOAE test, a follow-up was recommended at the age of 9–12 months (the results obtained at that time are summarised below under the term “1-year follow-up”).

Neonates who passed both, the AABR and TEOAE screenings, had no further scheduled follow-ups unless they had risk factors for late-onset hearing loss, such as congenital CMV infection.

The severity of HL was assessed by diagnostic ABR, as mentioned above, and classified according to the American Speech-Language-Hearing Association (ASHA): 26–40 dB nHL was defined as mild, 41–55 dB nHL as moderate, 56–70 dB nHL as moderately severe, 71–90 dB nHL as severe and > 90 dB nHL as profound HL [12].

In addition to the audiometric results and findings of the ENT examination at the first presentation and follow-ups, the following parameters were obtained from the chart review: age in days, sex, general medical history, family history, and risk factors for hearing disorders (congenital CMV infection, aminoglycoside treatment, positive family history, syndromes, non-syndromal craniofacial anomalies, perinatal complications such as premature birth, hyperbilirubinemia, low birth weight, mechanical ventilation, neonatal intensive care > 48 h). For further assessment of the course of hearing and treatment of hearing disorders (after the 1-year follow-up), data from the follow-up examinations until 12/2022 were retrieved from medical records, if available. The results were summarised below under the term “long-term” follow-up data.

Data analysis focussed on results from newborns, who passed the AABR screening, but failed the TEOAE test (AABR+/TEOAE-) in at least one ear. For comparative analysis, the remaining cases were analysed and summarised into four subgroups according to the results of the screening tests: AABR+/TEOAE+ , bilateral AABR-/TEOAE-, unilateral AABR-/TEOAE-, and AABR-/TEOAE+ .

This retrospective study was performed in accordance with the Declaration of Helsinki, and approved by the local ethics committee (project number 23–0248) and the data protection commissioner. After collection, all data were anonymised prior to analysis.

Statistical analysis was performed using SigmaStat software (Jandel Corp., San Rafael, CA, US) software. For descriptive statistics, we used mean values with standard deviation (SD) and median values with ranges. To compare the distribution of categorical data among different groups, a chi-square test was performed. A *p*-value ≤ 0.05 was considered significant.

## Results

Data were obtained from a total of 1095 infants (2190 ears). 253 newborns (23%) failed TEOAE but passed AABR screening tests (AABR+/TEOAE-) in 352 ears. In the remaining newborns, the following constellations of AABR/TEOAE were found: AABR+/TEOAE+ bilaterally in 433 neonates (40%), AABR-/TEOAE- bilaterally in 298 (27%), AABR-/TEOAE- unilaterally in 105 (10%) and AABR-/TEOAE+ in 6 (0.5%). Table 1 summarises the demographic data and clinical characteristics of the 253 children with AABR+/TEOAE- compared to all other children with different constellations of AABR and TEOAE.

**Table 1:** Demographic and clinical characteristics of all 1095 enrolled infants

	AABR+/TEOAE- uni- or bilateral *n* = 253 (23%)	AABR+/TEOAE+ bilateral *n* = 433 (40%)	AABR-/TEOAE- bilateral *n* = 298 (27%)	AABR-/TEOAE- unilateral *n* = 105 (10%)	AABR-/TEOAE+ uni- or bilateral *n* = 6 (0.5%)
Gender: male	150 (59%)	254 (59%)	174 (58%)	67 (64%)	5 (83%)
Female	103 (41%)	179 (41%)	124 (42%)	38 (36%)	1 (17%)
Mean age [days] ± SD	102 ± 51	105 ± 67	118 ± 56	110 ± 54	90 ± 57
Median age [days]	95	88	107	101	85
Range [days]	[6–211]	[9–214]	[24–209]	[8–210]	[33–176]
0 risk factor	145 (57%)	319 (74%)	126 (42%)	66 (62%)	1 (17%)
1 risk factor	75 (30%)	87 (20%)	116 (39%)	23 (22%)	3 (50%)
> 1 risk factor	33 (13%)	27 (6%)	56 (19%)	17 (16%)	2 (33%)
Type A tympanogram	59 (23%)	361 (83%)	119 (40%)	48 (46%)	4 (67%)
Season of 1st consult					
October to March	136 (55%)	208 (48%)	151 (51%)	49 (47%)	1 (17%)
April to September	117 (45%)	225 (52%)	147 (49%)	56 (53%)	5 (83%)

### Audiometric follow-up data

Follow-up diagnostics were recommended at the age of 12 months for children in the AABR+/TEOAE- group. In cases of persistent abnormal findings at the 1-year follow-up, a diagnostic ABR was scheduled.

**Fig. 1:**
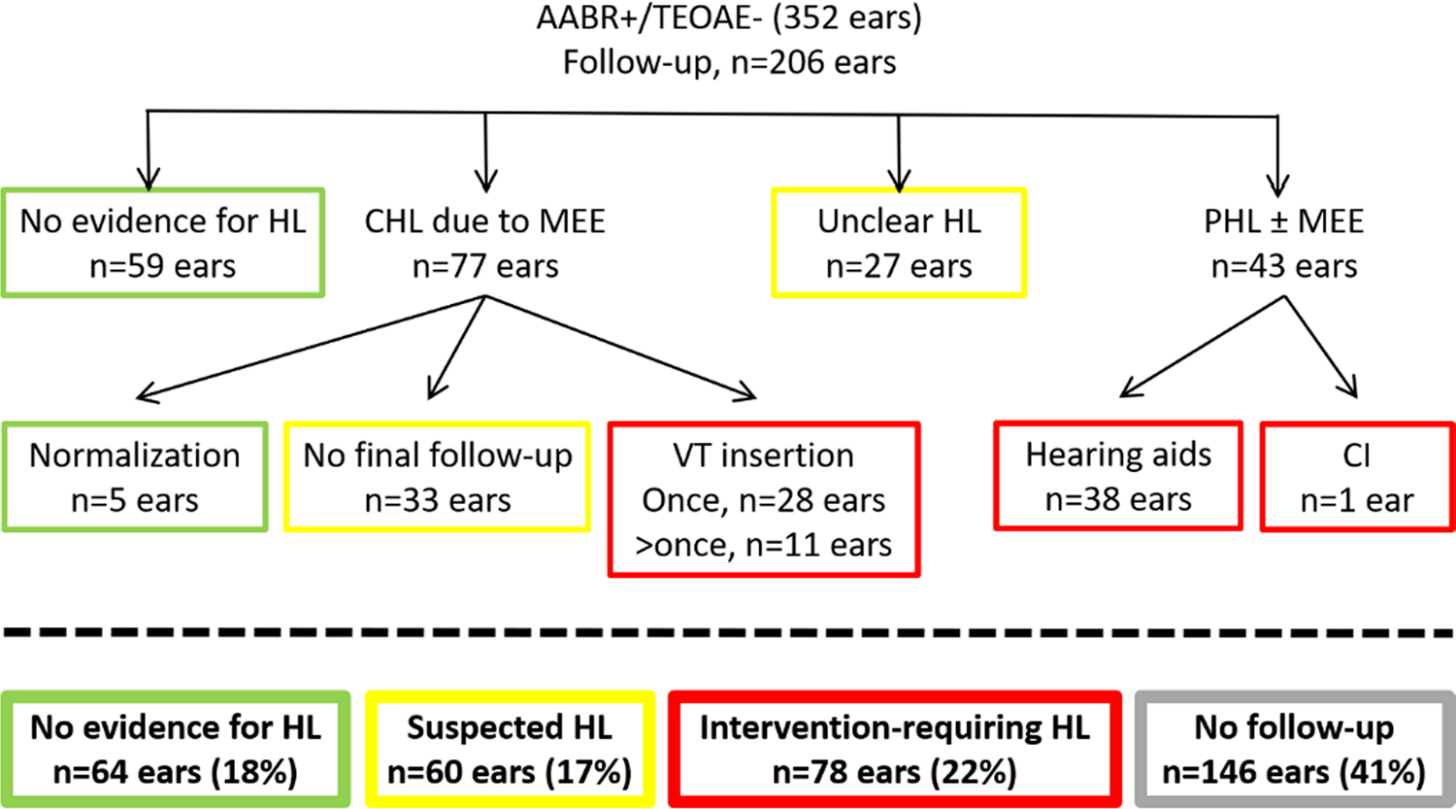
Long-term audiometric follow-up of 352 ears with AABR+/TEOAE-: The first row shows the results of the 1-year follow-up; the second row shows the findings obtained from further follow-ups. The last row summarises all results with respect to hearing loss (HL) (data from four ears are missing in the bottom row as they did not fit into the four subgroups)

Figure 1 summarises the follow-up results given per ear as the screening result of AABR+/TEOAE- was not always found in both ears of each infant of group AABR+/TEOAE-.

As shown in Fig. 1, 146 ears with AABR+/TEOAE- did not return to a 1-year follow-up. Of the remaining 206 ears, only 64 (31%) achieved audiometric results without evidence of HL during long-term follow-up.

The remaining 69% of ears returning to follow-up had either confirmed HL in 78 ears (requiring cochlear implantation, supply with a hearing aid, and/or insertion of ventilation tubes) or suspected HL in 60 ears. Among the 60 ears, 27 were classified as “unclear HL”, as they had no TEOAE detectable at the 1-year follow-up, but did not return to the next follow-up scheduled for diagnostic ABR. Therefore, differentiation between the type of HL (conductive vs. sensory vs. combined) and its severity was not possible in this small group of patients. In the remaining patients with suspected HL, scheduled diagnostic ABR was performed and showed conductive HL due to middle ear effusion (MEE). However, these children did not return for further follow-up at our department. Consequently, no information is available on the treatment or spontaneous clearance of MEE. In the last row of Fig. 1, data of four ears (1.9%) were excluded, as they did not fit into the four sub-groups: they had mild sensorineural HL, but refused any treatment, such as hearing aids.

Among the 253 neonates, 98 were affected by AABR+/TEOAE- on both ears. The remaining 155 infants showed AABR+/TEOAE- in only one ear, with AABR+/TEOAE+ in the contralateral ear in 86 infants, and AABR-/TEOAE- in the contralateral ear in 69 infants. Therefore, 184 neonates (17% of the whole study cohort of 1095 newborns) would not have been advised to return for follow-up, if the UNHS criteria had been applied which disregard absent TEOAE in the presence of a “pass” in the AABR screening.

Overall, 50 infants with abnormal results in the follow-up would have been monitored anyway due to a “refer” in the AABR screening on the contralateral ear. The remaining 55 infants with persistent HL at follow-up, however, would not have been further monitored within the UNHS protocol, as they achieved a “pass” in the AABR screening on both ears. Consequently, without TEOAE measurements, 55 children (5% of the total group under study) with persistent HL requiring therapy would have been underdiagnosed.

Table 2 shows the “long-term” follow-up data of the whole study group of 1095 neonates. The loss to follow-up varied greatly among the five cohorts as did the approximate follow-up period at our centre. Children who did not pass the AABR screening test, were obligated by the official UNHS survey to maintain further appointments which resulted in a high follow-up rate. Newborns of the AABR+/TEOAE- group were advised to follow-up voluntarily, resulting in a 40% loss due to lack of follow-up. In neonates with normal audiometric findings (AABR+/TEOAE+), follow-up was recommended only in the presence of risk factors for late-onset HL (e.g. congenital CMV infection). Of the 46 neonates who passed the AABR and TEOAE screening, 31 returned for follow-up due to such risks. The remaining 15 infants returned because of the clinical suspicion of HL or speech development disorders.

**Table 2:** Long-term audiometric follow-up of all 1095 enrolled infants

	AABR /TEOAE- *n* = 253	AABR+/TEOAE+ *n* = 433	AABR-/TEOAE- *n* = 298	Unilateral AABR-/TEOAE- *n* = 105	AABR-/TEOAE+ *n* = 6
Lost to follow-up	99 (39%)	387 (89%)	14 (5%)	7 (7%)	1 (17%)
Neonates with follow-up	154 (61%)	46 (11%)	284 (95%)	98 (93%)	5 (83%)
Mean period of follow-up [years]	1.35	1.87	1.76	1,07	1.25
Median period of follow-up [years]	0.70	1.72	1.28	0.87	0.74
Range [years]	[0.26–5.36]	[0.3–4.73]	[0.31–5.62]	[0.29–4.86]	[0.29–4.39]
Audiometric result at follow-up					
Permanent hearing loss					
Sensorineural hearing loss	32 (21%)	1 (2%)	146 (51%)	36 (39%)	3 (60%)
Conductive hearing loss	0 (0%)	0 (0%)	3 (1%)	5 (5%)	0 (0%)
Combined hearing loss	0 (0%)	0 (0%)	7 (2%)	0 (0%)	0 (0%)
Transient hearing loss					
MEE with hearing loss	55 (36%)	12 (26%)	98 (35%)	26 (26%)	0 (0%)
Normal hearing	49 (32%)	33 (72%)	29 (10%)	30 (30%)	2 (40%)
Unclear hearing loss	18 (12%)	0 (0%)	1 (0.4%)	1 (1%)	0 (0%)

### Permanent hearing loss

The long-term prevalence of permanent hearing loss (PHL), which was calculated upon all follow-up data, was 21% among AABR+/TEOAE- children compared with 54% in the AABR-/TEOAE- group, 44% in the unilateral AABR-/TEOAE- group and 60% in the AABR-/TEOAE+ group. Among children who passed AABR and TEOAE screening within the UNHS, one child (2%) acquired PHL despite AABR+/TEOAE+ .

**Fig. 2:**
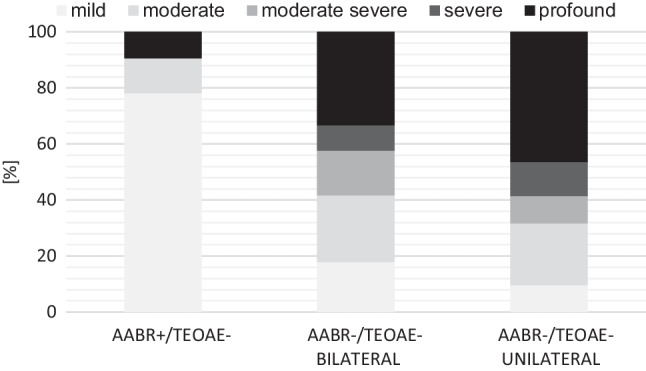
Severity of PHL assessed by diagnostic ABR in the different cohorts (PHL in neonates with AABR-/TEOAE+ and AABR+/TEOAE+ is not displayed due to the small sample sizes)

The severity of PHL assessed by diagnostic ABR differed between the groups, as shown in Fig. 2 (PHL in neonates with AABR-/TEOAE+ and AABR+/TEOAE+ is not shown due to the small sample size).

Most neonates in the AABR+/TOEAE- group showed mild PHL, in contrast to the other cohorts. However, three cases of profound HL and six cases of moderate HL were also observed.

### Middle ear effusion

The majority of AABR+/TEOAE- neonates presented an abnormal tympanogram at first consultation (77 vs. 37% in all other neonates; *p* < 0.001). In group-to-group comparison, an abnormal tympanogram was present significantly more in children with AABR+/TEOAE- than in any other AABR/TEOAE constellation (*p* < 0.05).

As MEE is the most common cause of disturbed middle ear ventilation and is often attributed to infectious otitis, we questioned whether the season affects the prevalence of AABR+/TEOAE-. Although infectious otitis occurs more frequently in winter, AABR+/TEOAE- is not predominantly observed from October to March, as shown in Table 1.

**Fig. 3:**
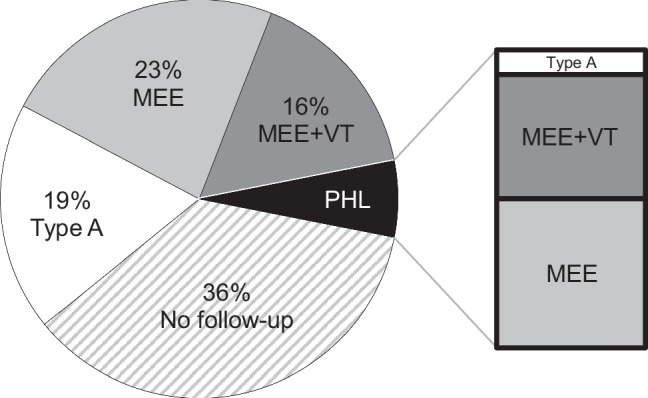
Outcome at 1-year follow up in 194 AABR+/TEOAE- infants with initially abnormal tympanogram. (Abbreviations: MEE—middle ear effusion; MEE + VT—middle ear effusion with insertion of ventilation tube; PHL—permanent hearing loss; Type A—peaked 1000 Hz-probe tone tympanogram)

Figure 3 displays the outcome at 1-year follow-up in all 194 AABR+/TEOAE- neonates with abnormal tympanograms at the initial consultation.

At 1-year follow-up, MEE was detected in 87 newborns out of 124 returning for follow-up after an initial abnormal tympanogram, resulting in a spontaneous MEE clearance of 30% within the first year of life. In 36 cases (30%), VT were inserted due to MEE. None of the families affected by persistent MEE reported experiencing HL in their daily lives. Overall, 6% of AABR+/TEOAE- neonates had PHL concomitant with MEE.

**Fig. 4:**
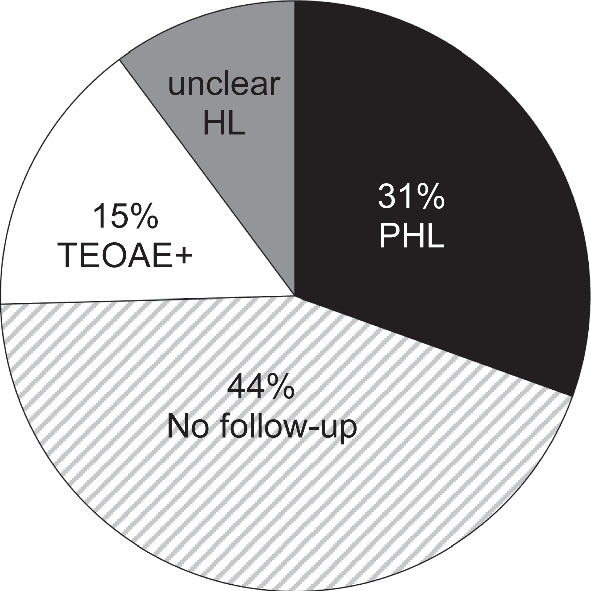
Long-term follow-up outcome of all 59 AABR+/TEOAE- newborns with tympanogram type A at initial consultation. (Abbreviations: HL—hearing loss; PHL—permanent hearing loss)

Figure 4 shows the long-term follow-up outcomes of all 59 AABR+/TEOAE- newborns with tympanogram type A at the initial consultation.

As demonstrated in Figs. 3 and 4, conductive HL solely due to MEE was the most frequent pathological finding in the AABR+/TEOAE- neonates. PHL is another reason for AABR+/TEOAE- constellation, which is rarely combined with MEE.

### Standard tympanometry vs. high-frequency tympanometry in neonates

Owing to the low rate of spontaneous MEE clearance and the high prevalence of MEE in AABR+/TEOAE- newborns, it is important to avoid overlooking MEE. Therefore, we analysed the validity of standard tympanometry at 226 Hz, which is the only tympanometry available in many otolaryngological practices.

Of the 1095 newborns, 84 underwent myringotomy within the first six months of life and had concomitant tympanometry with 1000 Hz- and 226 Hz-frequency tone probes preoperatively. The intraoperative myringotomy finding allowed a verification of the preoperative tympanometry result. The most common reason for early intervention was cleft surgery with simultaneous myringotomy due to suspected MEE (42 cases), followed by the suspicion of bilateral profound HL (32 cases) leading to diagnostic myringotomy within the diagnostic work-up.

According to the intraoperative findings in all 84 infants (151 ears) undergoing myringotomy, the accuracy of preoperative tympanometry with 1000- and 226 Hz-tone was calculated (Table 3).

**Table 3:** Comparison of tympanometry with 226 Hz- and 1000 Hz-tone probe

	226 Hz		1000 Hz		Sensitivity	226 Hz	1000 Hz
	Type A	Abnormal	Type A	Abnormal		0.44	0.96
No MEE intraop	43	3	41	4	Specifity	0.93	0.91
MEE intraop	59	46	4	102	Pos. pred. value	0.94	0.96
All tympanograms other than type A were classified as abnormal					Neg. pred. value	0.42	0.91
					Accuracy	0.59	0.95

### Prevalence of risk factors for hearing loss

Among the 1,095 newborns, 40% presented at least one risk factor for early hearing disorders. In AABR+ /TEOAE- neonates, the prevalence of risk factors was similar (43%), but significantly lower than in AABR+/TEOAE + newborns (26%, *p* < 0.0001).

**Fig. 5:**
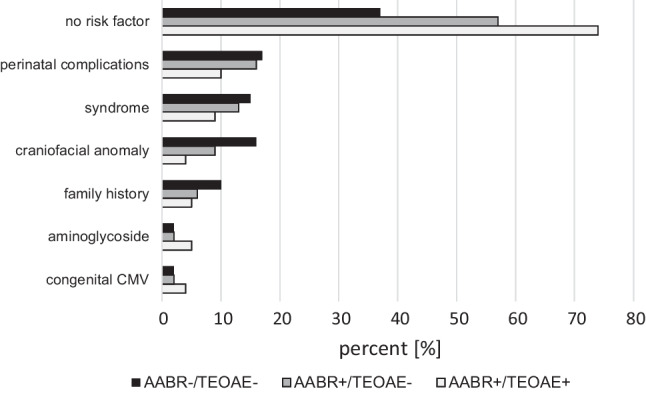
Distribution of risk factors in different cohorts (AABR+/TEOAE-, AABR+/TEOAE+ and AABR-/TEOAE-). 100% corresponds to the total number of patients within each cohort (AABR+/TEOAE-, *n* = 253; AABR+/TEOAE+ , *n* = 433; and AABR-/TEOAE-, *n* = 298)

Figure 5 shows the distribution of risk factors among different cohorts (AABR+/TEOAE- vs. AABR+/TEOAE + vs. AABR-/TEOAE-).

Among AABR+/TEOAE- newborns, the profile of risk factors resembled the profile in AABR-/TEOAE- neonates more than that in AABR+/TEOAE+ newborns. A total of 65 of 99 neonates in the AABR+/TEOAE- group with at least one risk factor showed HL at the 1-year follow-up (66%) compared to 49 of 154 AABR + /TEOAE- neonates without risk factors (32%), resulting in a relative risk (RR) of 1.77 (95% confidence interval (CI): 1.333 to 2.354) and an odds ratio (OR) of 2.79 (95% CI: 1.681 to 4.644) for the presence of risk factors and the incidence of HL. Long-term results were similar with 88 cases of HL (52 with and 36 without risk factors) and 49 cases of normalised results (14 with and 35 without risk factors), yielding a RR of 1.55 (95% CI: 1.197 to 2.018) and an OR of 3.61 (95% CI: 1.703 to 7.656). In the AABR-/TEOAE- cohort, however, the association between risk factors and the incidence of HL was weak (RR 1.06, 95% CI 0.978 to 1.157; OR 1.80, 95% CI 0.832 to 3.911).

### Reproducibility of the data

To investigate whether the results of the present study are representative, reproducible and unaffected by outliers, the data were analysed separately for each year of the observation period. As shown in Table 4, the prevalence of AABR+/TEOAE- only varied between 20% minimum (in 2020 and 2021) and 27% maximum (in 2017) throughout the 5 year-period. Further, the percentage of neonates with AABR+/TEOAE- who showed HL at follow-up (MEE and PHL) varied only little from year to year (average 36%, minimum 30% and maximum 42%). Furthermore, the number of patients, mean age, male predominance, and absence of risk factors remained stable throughout the 5 year-period of observation.

**Table 4:** Year-per-year comparison of demographic and clinical data

	2017	2018	2019	2020	2021	Total (2017–2021)
Total number of patients	232	233	205	203	222	1095
Result of hearing screening						
AABR + /TEOAE-	63 (27%)	55 (24%)	52 (25%)	40 (20%)	43 (20%)	253 (23%)
HL at follow-up	19 (30%)	22 (40%)	19 (37%)	12 (30%)	18 (42%)	90 (36%)
AABR + /TEOAE +	84 (36%)	103 (44%)	82 (40%)	80 (39%)	84 (36%)	433 (40%)
AABR-/TEOAE-	67 (29%)	57 (24%)	51 (25%)	57 (28%)	66 (29%)	298 (28%)
unilat. AABR-/TEOAE-	17 (7%)	15 (6%)	19 (9%)	26 (13%)	28 (12%)	105 (9%)
AABR-/TEOAE +	1 (0.4%)	3 (1%)	1 (0.5%)	0 (0%)	1 (1%)	6 (1%)
Gender						
Male	138 (59%)	136 (59%)	126 (61%)	123 (61%)	127 (57%)	650 (60%)
Female	94 (41%)	97 (41%)	79 (39%)	80 (39%)	95 (43%)	445 (41%)
Age						
Mean [days] ± standard dev	104 ± 52	106 ± 56	107 ± 54	99 ± 57	101 ± 51	104 ± 54
Median [days]	96	99	99	87	93	94
Range [days]	11–214	6–208	8–211	11–207	9–209	6–214
No risk factor	136 (59%)	139 (60%)	125 (61%)	123 (61%)	134 (60%)	657 (60%)

## Discussion

As any screening tool, UNHS must maintain a balance between maximum sensitivity and specificity. Maintaining a low referral rate is probably why newborns who fail TEOAE in the first step of UNHS, but pass AABR in the second step (AABR+/TEOAE-), have no further officially recommended follow-up and are thereby treated identically to neonates who pass both, TEOAE and AABR (AABR+/TEOAE+).

The present study characterised a cohort of 253 newborns with AABR+/TEOAE- and compared their data with those of 842 newborns with screening results other than AABR+/TEOAE-. As shown in Table 4, the data varied only slightly from year to year within the 5 year-observation period. The total number of patients decreased between 2019 and 2020. In 2020, this decline could be attributed to the onset of the Covid-19 pandemic, which, at least during the first lockdown period, may have discouraged families from consulting larger hospitals. Many studies from different countries have shown a significant overall reduction in healthcare use by 2020 with respect to paediatric illnesses [13] and ENT consultations [14, 15]. As the total number of patients had already decreased in 2019, the reason could also be explained by a decline in birth numbers registered from 2018 to 2019 by the Federal Statistical Office. The same institution reported only a slight male predominance among children born between 2017 and 2021 (51% boys, 49% girls). Our cohort of 60% boys and 40% girls confirmed that boys were more affected by UNHS failure than girls. This sex predominance has also been described in other studies and is partially attributable to the sex demographics of most neonatal intensive care units [16].

The analysis revealed several distinctive characteristics in neonates with AABR+/TEOAE-, which demonstrates that newborns who fail TEOAE screening despite passing AABR tests deserve closer attention: they have a high prevalence of (i) disturbance in middle ear ventilation at the initial consultation, (ii) persistent MEE at the 1-year follow-up, (iii) PHL not caused by MEE as a long-term outcome, and (iv) risk factors for HL. Further, the association between the presence of risk factors and the incidence of HL was stronger in the group AABR+/TEOAE- than in neonates who failed both AABR and TEOAE screening.

### Hearing loss due to middle ear effusion

Initially, 194 (77%) of the 253 newborns with AABR+/TEOAE- showed an abnormal (non-type A) tympanogram for the 1000 Hz probe tone indicating a middle ear ventilation impairment. Given the difficulties in ear microscopy in neonates, it can be questioned, if all abnormal initial tympanograms were due to disturbed middle ear ventilation: collapsed ear canals as well as ear wax might have partially affected the initially high prevalence. Nevertheless, this confounding factor also applied to the other 842 neonates included in the control groups. Among the 124 AABR+/TEOAE- neonates who had an initially abnormal tympanogram and who returned for follow-up, 36 (29%) achieved a normalisation of the tympanogram in the course of further follow-ups.

The 29% rate of spontaneous MEE clearance was similar to an earlier finding by Karawani and colleagues, who recorded a resolution rate of 26% in 46 newborns in Israel [17]. The prognosis of acquired MEE, which is mainly attributed to infectious otitis, is reportedly much more favourable [18]. Therefore, the present data confirm that congenital MEE resolves at a lower rate than non-congenital MEE.

In contrast to acquired MEE in toddlers, which occurs more frequently during winter times [19], no significant correlation between MEE and season was observed in our neonatal cohort. The tympanometric condition of neonates is less affected by season-dependent infections, but rather by small anatomical structures as well as by the differing composition of congenital MEE.

It appears that in many newborns, the absence of TEOAEs is the only indicator for MEE within UNHS: The screening is not only conducted by paedaudiologists, but also by otolaryngologists, paediatricians, and nurses, who do not have access to high-frequency tympanometry and who are not always highly experienced in difficult neonatal ear microscopy. Our comparative analysis of standard- and high-frequency tympanometry in relation to intraoperative findings at myringotomy in 84 cases demonstrated that tympanometry with a 226 Hz-frequency probe tone does not allow valid middle ear assessment in neonates. This is in line with other studies [20–23] which correlated tympanometry with otoscopic examination, ear microscopy, or various audiometric tests, such as diagnostic ABR or TEOAE, rather than with intraoperative findings.

Without follow-up, which was only initiated due to the absent TEOAE in the screening, the time-to-diagnosis would have been much longer, as all parents of children with isolated MEE reported at follow-up consultation not noticing any signs of HL in their child. Similar results were reported in an earlier survey from the US which showed that mild HL in children without risk factors was first suspected by parents at 15 months of age [24].

### Permanent hearing loss

In 21% of the AABR+/TEOAE- newborns returning for follow-up, permanent sensory or conductive HL was diagnosed. Among newborns who passed TEOAE and AABR screening tests, the prevalence of PHL (either sensory or conductive) was significantly lower (2%) in long-term. However, the low rate of follow-up among children with AABR+/TEOAE+ must be considered (11 vs. 60% in AABR+/TEOAE-). But even among the 45 children returning to a follow-up due to risk factors or a clinical suspicion of HL, only one child (2%) presented an assumingly newly acquired permanent HL and 12 showed conductive HL due to MEE (27%). The finding that only one infant out of the 1,095 analysed neonates developed newly acquired permanent HL (despite AABR+/TEOAE+) supports the suggestion that newly acquired HL is rare in childhood. According to the literature, the rate of significant HL among school-age children is up to twice that of newborns [25]. Our data suggest that some of these assumed acquired/late-onset hearing disorders might already have existed at birth, but might have been underdiagnosed by UNHS due to the initially mild severity.

In the majority of children in the AABR+/TEOAE- group with HL, the severity of HL was mild as expected. However, mild HL carries the risk of not being noticed by parents in everyday life. In particular if the mild HL is congenital, parents are used to their child’s slightly reduced hearing since birth and consider it adequate according to the passed UNHS. Consequently, mild congenital HL might be easily overlooked. Without the recommendation for further audiometric follow-up due to abnormal TEOAE in UNHS, the time-to-diagnosis would probably have been delayed, not only in cases with persistent MEE, but also in children affected by PHL.

Nowadays, there is a relatively broad consensus that even mild HL affects auditory and speech development in the paediatric population. In 2011, a meta-analysis showed that MEE with elevated hearing thresholds was associated with lasting speech perception and auditory deficits [26]. Following this meta-analysis, many other studies have confirmed that deficits in auditory abilities [27, 28] and speech processing [29] can be observed at later ages, even after years when MEE has resolved and hearing thresholds have returned to normal, probably because of changes in the central auditory nervous system [30].

### Prevalence of risk factors

Of all the neonates affected by PHL (regardless of the AABR/TEOAE status), only 52% had known risk factors. This result confirms previous studies [16, 31] demonstrating that only approximately half of the newborns with PHL had risk factors—the rationale of any UNHS opposed to targeted testing of high-risk neonates.

However, among the AABR+/TEOAE- newborns, the prevalence of risk factors was significantly higher than that in the AABR+/TEOAE+ cohort, especially with respect to non-syndromic craniofacial anomalies. According to the literature, syndromes associated with HL have the highest association with incidence of HL, followed by congenital anomalies of the head and neck [16].

The overall association between the presence of risk factors and the incidence of HL at the 1-year follow-up was stronger among infants with AABR+/TEOAE- compared to children of the AABR-/TEOAE- group.

This finding emphasises that neonates with AABR+/TEOAE- are a special pathological entity deserving closer attention than newborns with AABR+/TEOAE+ screening results, especially in the presence of risk factors. Given the high prevalence of intervention-requiring HL among infants in the AABR+/TEOAE- group, taking care of affected children differently than neonates in the AABR+/TEOAE+ group is meaningful.

### Limitations of the study

The present study has some limitations, mainly owing to its retrospective design. First, the follow-up rates differed widely across cohorts. It is possible that the AABR+/TEOAE- children lost to follow-up experienced normalisation of the results proven elsewhere. However, even under the optimistic assumption, that all 99 lost to follow-up AABR+/TEOAE- neonates experienced normalisation, there was a substantial number of 87 infants (34%) among 253 AABR+/TEOAE- neonates with proven hearing impairment and further 18 infants (7%) with high suspicion of hearing impairment.

Further, it is conceivable that our study missed AABR+/TEOAE+ children who developed hearing disorders, but sought advice in other institutions. Especially with respect to MEE, many families might turn to their family ENT doctors nearby, rather than to our tertiary referral centre. However, with respect to permanent hearing disorders requiring amplification, it appears quite unlikely that our study missed many cases due to loss of follow-up: as regulatory demands restrict the prescription of hearing aids in children to doctors specialised in paediatric audiology, it seems probable that children affected by acquired HL would have returned to our tertiary referral centre. Nevertheless, it cannot be excluded that our study missed children in the AABR+/TEOAE+ group who developed hearing disorders at a later point in time, either due to MEE or acquired or progressive sensorineural hearing loss. Hence, owing to the retrospective study design, children with acquired HL might have been underrepresented in the AABR+/TEOAE+ group.

Second, our data were based on newborns who were referred to our centre because of failure in the initial UNHS conducted elsewhere (e.g., the obstetric clinic). Therefore, there was an additional selection bias towards pathological cases. in this context, a tertiary referral centre provides optimal diagnostic facilities and experienced staff with longer time windows for diagnostics, which could have influenced the present data. Consequently, the conclusions drawn from our data cannot be applied to the total population of neonates or all types of hospitals. However, our study population corresponds to the collective of patients seen by many otorhinolaryngologists, who are most frequently not involved until neonates fail initial UNHS performed in obstetric clinics.

A further limitation of our study is that diagnostic ABR was only performed in AABR+/TEOAE- infants when they did not have detectable TEOAE at the 1-year follow-up. This time delay resulted in 18 unclear cases among the 253 AABR+/TEOAE- infants, as they returned to follow-up at the age of 9–12 months, but did not return to diagnostic ABR despite pathologic findings at the first follow-up. It is desirable to conduct diagnostic ABR not only in newborns with failure in both screening tests (TEOAE and AABR) but also in each infant who fails TEOAE screening despite passing the AABR test. However, in Germany, there is limited capacity for this highly specialised diagnostic tool. Providing diagnostic ABR to all AABR+/TEOAE- neonates would result in a longer waiting time leading to an unacceptable delay in the diagnosis of moderate-to-profound hearing disorders which require treatment as soon as possible.

## Conclusion

Despite the limitations of the retrospective study design, our data demonstrate that failed TEOAE screening, despite passed AABR test within the UNHS is associated with a high prevalence of HL, either due to persistent MEE or permanent sensory or conductive HL. The rate of spontaneous clearance of congenital MEE is lower than that of acquired MEE. Therefore, a substantial number of ventilation tubes is required. Consistent with an increased prevalence of HL, the prevalence of risk factors is significantly higher in neonates with AABR+/TEOAE- compared to newborns passing both screening tests, AABR and TEOAE. Apart from a few cases with progressive HL, most affected infants experience mild HL. However, even mild HL may influence speech development at later ages causing long-term effects. Therefore, we consider measurement of TEOAE as a valuable addition to AABR registration in newborns, particularly in the presence of risk factors. In cases of failed TEOAE screening, despite passed AABR test, audiometric follow-up is recommended to avoid overlooking hearing impairments.
